# Oxidative Stress and Mitochondrial Damage in Neurodegenerative Diseases: From Molecular Mechanisms to Targeted Therapies

**DOI:** 10.1155/2020/1270256

**Published:** 2020-05-04

**Authors:** Giovanna Cenini, Ana Lloret, Roberta Cascella

**Affiliations:** ^1^Institut für Biochemie und Molekularbiologie, University of Bonn, 53115 Bonn, Germany; ^2^Department of Physiology, Faculty of Medicine, University of Valencia, Avda, Blasco Ibañez17, 46010 Valencia, Spain; ^3^Department of Experimental and Clinical Biomedical Sciences, Section of Biochemistry, University of Florence, 50134 Florence, Italy

A growing body of evidence suggests the alteration of the reduction-oxidation (redox) homeostasis in the brain grown with the increasing of the age. The brain is composed of highly differentiated cells that populate different anatomical regions and requires about 20% of body basal oxygen for its functions [1]. Thus, it is not surprising that oxidative stress, as well as alterations in brain energy metabolisms, have been implicated in the pathogenesis of several neurodegenerative diseases, including Alzheimer's disease (AD), Parkinson's disease (PD), and amyotrophic lateral sclerosis (ALS). These neurodegenerative disorders are typically characterized by the progressive loss of neuronal cells and compromised motor or cognitive functions. It has been shown that neuronal cells are particularly vulnerable to oxidative damage due to their high polyunsaturated fatty acid content in membranes, high oxygen consumption, and weak antioxidant defence. Cellular energy is mainly produced via oxidative phosphorylation taking place within mitochondria, which are crucial organelles for numerous cellular processes, such as energy metabolism, calcium homeostasis, lipid biosynthesis, and apoptosis [2, 3]. Glucose oxidation is the most relevant source of energy in the brain because of its high rate of ATP generation needed to maintain neuronal energy demands [1]. Thus, neurons rely almost exclusively on the mitochondria, which produce the energy required for most of the cellular processes, including synaptic plasticity and neurotransmitter synthesis [4].

This special issue contributes to original articles that highlight and unravel mechanisms by which oxidative stress and mitochondrial damage are implicated in neurodegenerative diseases and provide new strategies that may counteract these pathological processes.

The manuscript by A.A. Abubaker et al. highlights the importance of NADPH oxidase activation and platelet oxidative responses in the prothrombotic responses induced by A*β*1-42, which is the *β*-amyloid peptide accumulating in the brain of Alzheimer's and Cerebral Amyloid Angiopathy (CAA) patients. In addition to giving us some direction in the elucidation of the molecular mechanisms underlying platelet activation by *β*-amyloid peptides, this study suggests a potential therapeutic opportunity aiming at limiting the vascular component of Alzheimer's disease by targeting NADPH oxidase activity.

C.C. Cai et al. provides the first evidence that glycine, a common substance present in numerous biomolecules, attenuated hypoxic-ischemic injury in neurons or nervous systems by decreasing mitochondria–mediated autophagy through regulating the AMPK pathway.

J. Budziosz et al. investigated the effects of low-frequency electromagnetic field (LFEMF) on the human body as electromagnetic sensitivity syndrome is commonly associated with the rapid development of wireless technologies. Several researchers have emphasised that exposure to EMF might also cause increased ROS production and lead to oxidative stress, which has been implicated in the pathogenesis of neurodegenerative diseases. Regardless, the researchers did not find any differences in lipid peroxidation, total oxidant status, and antioxidant systems between the experimental and control groups, suggesting that LFEMF did not affect oxidative stress in the investigated brain structures.

K-I Tanaka et al. examined the effect of Ni^2+^ on Zn^2+^-induced neurotoxicity, focusing on the endoplasmic reticulum (ER) stress response, and found that carnosine (an endogenous peptide) attenuated Ni^2+^/Zn^2+^-induced neuronal cell death and ER stress occurring before cell death. Based on their results, Ni^2+^ treatment significantly enhances Zn^2+^-induced neuronal cell death by priming the ER stress response. Thus, compounds that decrease the ER stress response, such as carnosine, may be beneficial for neurological diseases.

The role of mitochondrial quality control (MQC) was investigated by X. Jiang et al. This review focused on three main aspects, that is, mitochondrial biogenesis, mitochondrial dynamics, and mitochondrial autophagy showing how genetic and environmental factors result in PD pathogenesis by interfering with MQC pathways, thereby hopefully contributing to the discovery of novel potential therapeutic targets for PD.

J. Han et al. examined the effects of paraquat (PQ), an herbicide considered an environmental contributor to the development of PD, inducing dopaminergic neuronal loss through reactive oxygen species (ROS) production and oxidative stress by mitochondrial complex I. Their findings indicate that the inhibition of mitochondrial complex I with chloramphenicol (CP) protects dopaminergic neurons and may provide a strategy for preventing neurotoxin-induced PD.

Z. Wang et al. quantitatively pooled data on levels of blood oxidative stress markers in ALS patients from the literature using a meta-analytic technique. They showed significantly increased blood levels of 8-hydroxyguanosine, malondialdehyde, and advanced oxidation protein product as well as decreased glutathione and uric acid levels in the peripheral blood of ALS patients. Thus, this meta-analysis clarifies the oxidative stress marker profile in the blood of ALS patients and strengthens the clinical evidence that prooxidative imbalances contribute to ALS pathophysiology.

H-S. Lim et al. investigated the protective effects of Cicadidae Periostracum (CP), the cast-off skin of Cryptotympana pustulata Fabricius, on 1-8 methyl-4-phenyl-1,2,3,6-tetrahydropyridine- (MPTP-) induced PD in mice and investigated the underlying mechanisms of action, focusing on Nuclear receptor-related 1 protein (Nurr1), a nuclear hormone receptor implicated in limiting mitochondria dysfunction, apoptosis, and inflammation in the central nervous system and protecting dopaminergic neurons. They showed that CP might contribute to neuroprotective signalling by regulating neurotrophic factors primarily via Nurr1 signalling, neuroinflammation, and mitochondria-mediated apoptosis.

J.H. Cater et al. reviewed the ability of hypochlorite, an oxidant that is generated during inflammation, to regulate alpha-2-macroglobulin (*α*2M). This tetrameric protein is constitutively abundant in biological fluids and is involved in several biological processes, including the clearance of the A*β* peptide.

In the end, the role of mitochondrial oxidative stress in the aging process and neurodegenerative diseases has been further explored by G. Cenini et al. The review tried to summarize the molecular mechanisms involving mitochondria and oxidative stress in the aging process with the aim at identifying new strategies for improving a healthy and extending lifespan.
